# Promotion of Treg/Th17 balance in MRL/lpr mice by Jianpi-Zishen Formula via modulation of DNMT1-mediated Foxp3 methylation

**DOI:** 10.3389/fimmu.2025.1631631

**Published:** 2025-08-21

**Authors:** Ming Li, Lijun Pang, Yunfei Li, Junjie Chen, Shuangshuang Shang, Chuanbing Huang

**Affiliations:** ^1^ Department of Rheumatology, The First Affiliated Hospital of Anhui University of Chinese Medicine, Hefei, Anhui, China; ^2^ Center for Xin’an Medicine and Modernization of Traditional Chinese Medicine of Institute of Herbal Medicine (IHM), Hefei, Anhui, China

**Keywords:** Jianpi-Zishen Formula, systemic lupus erythematosus, DNA methylation, Dnmt1, Foxp3, Treg/Th17 rebalance

## Abstract

**Purpose:**

This study aimed to investigate whether Jianpi-Zishen Formula (JPZS) modulates the Treg/Th17 balance in MRL/lpr mice through regulation of DNA methyltransferase 1 (DNMT1)-mediated forkhead box P3 (Foxp3) methylation, and to elucidate its potential mechanism for improving immune homeostasis in systemic lupus erythematosus (SLE).

**Methods:**

Forty-eight female MRL/lpr mice were randomized into six groups (n=8/group): JPZS (low/medium/high doses), 5-aza-CdR (DNMT inhibitor), DC_517 (DNMT1 inhibitor), and model control. Eight C57BL/6 mice served as healthy controls. The mice were subjected to the corresponding intervention measures for eight weeks. The impact of JPZS on the disease progression of MRL/lpr mice was evaluated using enzyme-linked immunosorbent assay (ELISA) and serum biochemical parameters. Moreover, immunofluorescence staining and flow cytometry were employed to investigate alterations in the proportions of Tregs and Th17 cells. CD4^+^ T cells were isolated from the spleen for subsequent investigation, including quantitative real-time PCR, western blotting, and determination of DNA methylation levels. Furthermore, the enzymatic activity of CD4^+^ T cell-specific DNA methyltransferases was quantified using an EpiQuik DNMT detection kit.

**Results:**

JPZS significantly improved the disease development of MRL/lpr mice in a dose-dependent manner. Flow cytometry and immunofluorescence indicated JPZS promoted Treg/Th17 rebalancing. Research has found that *Foxp3* is at a high methylation level in CD4^+^ T cells of the model group, and the transcription level of *Foxp3* mRNA is downregulated; JPZS can downregulate *Foxp3* methylation levels of CD4^+^ T cells in the model group. Further research has found that the level of *Foxp3* methylation is closely related to Dnmt1 enzyme activity, and JPZS can downregulate Dnmt1 enzyme activity, thereby upregulating the transcription level of *Foxp3* mRNA.

**Conclusion:**

JPZS may restore Treg/Th17 balance in SLE via DNMT1-regulated *Foxp3* demethylation, suggesting an epigenetic mechanism for its immunomodulatory effects.

## Introduction

Systemic lupus erythematosus (SLE) is a prototypic B cell-driven autoimmune disease characterized by pathogenic autoantibody production, immune complex deposition, and multi-organ inflammation (1). While B cell hyperactivity is central to disease pathogenesis, emerging evidence underscores the critical role of T cell dysregulation in shaping aberrant humoral immunity (2). Regulatory T cells (Tregs), characterized by forkhead box P3 (Foxp3) expression, maintain peripheral tolerance through suppression of effector T cells and direct modulation of B cell responses via CTLA-4-mediated inhibition of CD40L signaling (3–6). Conversely, T follicular helper (Tfh) cells are programmed death receptor-1 (PD-1)+C-X-C motif chemokine receptor 5 (CXCR5)+CD4+ T cells that are involved in B cell differentiation and maturation in germinal centers of secondary lymphoid tissues (7, 8). Tfh cells promote pathogenic B cell responses in SLE (9). The imbalance between immunosuppressive Tregs and pro-inflammatory Th17 cells disrupts immune homeostasis, creating a permissive environment for autoreactive B cell expansion and autoantibody production in SLE (10, 11).

Epigenetic mechanisms, particularly DNA methylation, play pivotal roles in regulating T cell lineage commitment and functional stability (12). DNMT1, the canonical maintenance methyltransferase, ensures transmission of epigenetic information during mitosis by methylating hemimethylated CpG dinucleotides at replication foci (13). In regulatory T cells, DNMT1-dependent methylation of conserved non-coding sequences within the Foxp3 locus (e.g., CNS2 region) establishes a repressive chromatin architecture that fine-tunes Foxp3 expression levels (14). Aberrant DNMT1 activity in SLE has been shown to promote Foxp3 hypermethylation, leading to reduced Treg stability and compromised suppressive capacity (15, 16). Concurrently, IL-6-driven STAT3 activation in Th17 cells induces ten-eleven translocation (TET) enzyme downregulation, resulting in hypomethylation of RORγt and IL-17A loci that amplifies their pathogenic potential (17). This epigenetic dysregulation creates a vicious cycle exacerbating Treg/Th17 imbalance and autoimmune progression.

SLE pathogenesis is deeply rooted in the TCM concept of spleen-kidney yin deficiency, a syndrome characterized by disrupted Yin-Yang balance and multi-system immune dysregulation. Modern research elucidates this ancient paradigm: spleen yin deficiency impairs metabolic homeostasis and nutrient transformation, manifesting as fatigue and edema, while kidney yin depletion compromises genetic regulation and hormonal balance, linked to low-grade fever and photosensitivity (18, 19). Critically, yin deficiency drives a pro-inflammatory state via Th17/Treg imbalance, with elevated Th17 cells secreting IL-17 and reduced Treg cells losing immunosuppressive capacity—a phenomenon validated in SLE patients with active disease (20).

Jianpi-Zishen Formula (JPZS), a refined Liuwei Dihuang Wan, embodies the TCM principle of “strengthening spleen and nourishing kidney” through eight synergistic botanicals. Astragalus membranaceus enhances Treg stability via Foxp3 upregulation (21, 22), while Rehmannia glutinosa inhibits DNMT1 (23), correcting DNA hypomethylation observed in SLE T cells. Poria cocos suppresses IL-17 production, directly counteracting Th17-driven inflammation (24). Clinical trials demonstrate JPZS restores peripheral Treg/Th17 ratios and reduces SLEDAI scores, though molecular mechanisms remain under investigation (25, 26). However, the exact mechanism by which JPZS influences the Treg/Th17 balance has not yet been thoroughly investigated.

In summary, this study sought to clarify the therapeutic effects of JPZS in the MRL/lpr mouse model of SLE. By investigating the modulation of the Treg/Th17 balance and the regulation of *Foxp3* methylation through DNMT1, we hope to clarify the underlying mechanisms of TCM in autoimmune diseases and provide potential targets for therapeutic intervention.

## Materials and methods

### Preparation of JPZS extracts

**Figure 1:**
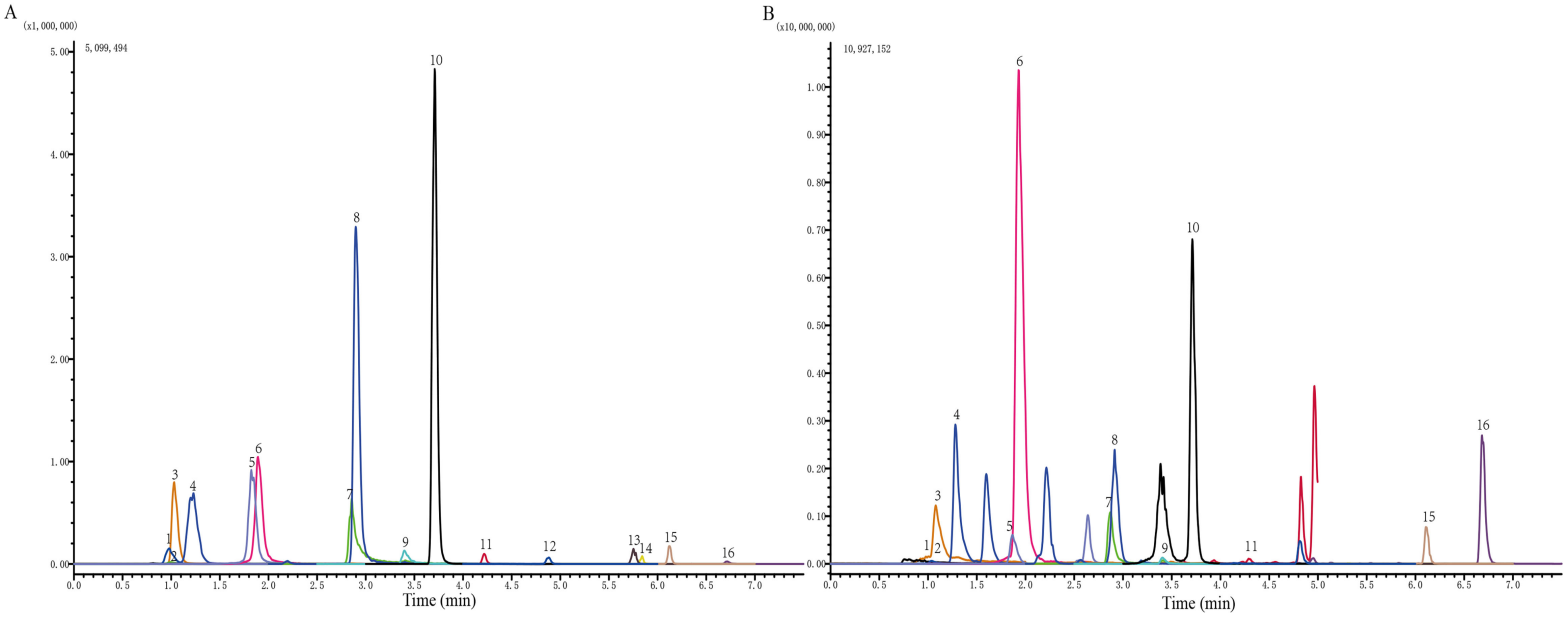
Identification of the chemical components of JPZS. **(A)** Standard product; **(B)** JPZS. The main components identified are as follows: 1. catechin, 2. gallic acid, 3. 5-hydroxymethylfurfural, 4. acteoside, 5. rutin, 6. hyperoside, 7. quercetin, 8. tiliroside, 9. kaempferol, 10. formononetin, 11. atractylenolide III, 12. astragaloside A, 13. dioscin, 14. atractylodin, 15. pachymic acid, and 16. atractylone.

Huangqi [*Astragalus membranaeus* (Fisch.) Bge.], Tusizi (*Cuscuta chinensis* Lam.), Baizhu (*Atractylodes macrocephala* Koidz.), Shudihuang (*Rehmannia glutinosa* Libosch.), Shanyao (*Dioscorea opposita* Thunb.), Fuling [*Poria cocos* (Schw.) Wolf], Fupenzi (*Rubus chingii* Hu), and Jinyingzi (*Rosa laevigata* Michx.) were purchased from Bozhou Medicine Company (batch number: 180609), Anhui, China. The JPZS formulation comprises eight types of processed Chinese medicinal herbs. First, a precise amount of the aforementioned Chinese medicinal herbs were meticulously weighed. Subsequently, the herbs were immersed in water at a volume 10-fold their weight and left to soak for 1 h. They then underwent two rounds of extraction using boiling water, with each round lasting 1.5 h. The resulting extracts from both rounds were combined and filtered. Finally, the filtered solution was concentrated to 2.522 g/mL of raw dry material using a rotary evaporator. The concentrated extract (2.522 g raw herb/mL) was aliquoted and stored at -80°C. High-performance liquid chromatography (HPLC) fingerprinting (**Figure 1**) was employed to ensure batch consistency for this study. Future investigations will incorporate quantitative standardization based on key marker compounds identified in the HPLC profile.

### High-performance liquid chromatography analysis of JPZS

The main constituents of the JPZS formulation were analyzed and quantified using HPLC. The preparation substances used for analysis included catechin (B21722), gallic acid (B20851), 5-hydroxymethylfural (B21382), acteoside (B20715), rutin (B20711), hyperoside (B20631), quercetin (B20257), tiliroside (B21587), kaempferol (B21126), formononetin (B20836), atractylenolide III (B20056), astragaloside A (B20564), dioscin (B1176), atractylodin (B20128), pachytic acid (B20400), and atractylone (B20129). These substances were obtained from Shanghai Yuanye Biotechnology Co., Ltd. at Shim-pack GIST C18 column (2×100mm, particle size 2.0 μm) using reverse phase HPLC. B methanol (0.01% formic acid) - A water (0.01% ammonium formate) was used as the mobile phase during the following gradient elution procedure for 14 min: 0 min, 50% B; 5 min, 100% B; 8 min, 100% B; 8.1 min: 50% B. Flow rate: 0.3 mL/min. With the column temperature at 40°C, the detection wavelength was adjusted to 218 nm.

### Animals and experimental design

A total of 48 SPF-grade female MRL/lpr mice and 8 SPF-grade female C57BL/6 mice (6 weeks old) were purchased from Shanghai Slac Laboratory Animal Co., Ltd. (Animal license number: SCXK [Hu] 2022-0004). All mice were housed in the animal facility of the First Affiliated Hospital of Anhui University of Chinese Medicine, where the temperature was maintained at 23 ± 2°C, humidity was kept between 40–60%, and a 12-hour light/dark cycle was followed. Adaptive feeding was conducted for 2 weeks before the start of the experiments. 48 MRL/lpr mice were randomly divided into six groups with eight mice: model group, low-dose JPZS group (6.305 g/kg, JPZS-L), medium-dose JPZS group (12.61 g/Kg, JPZS-M), high-dose JPZS group (25.22 g/kg, JPZS-H), 5-aza-CdR inhibitor group, and DC_517 inhibitor group, with eight mice in each group. eight female C57BL/6 mice were used as controls. JPZS group (Low, medium, and high doses) is administered with corresponding doses of JPZS, the control, model, 5-aza-CdR inhibitor, and DC_517 inhibitor groups were given the same volume of physiological saline solution, once a day for 8 weeks.

5-Aza-2-deoxycytidine (Selleck, cat. S1200) is a DNMT inhibitor while DC_517 (DAC; MCE, cat. HY-A0004) is a DNMT1 inhibitor. The 5-aza-CdR inhibitor group and DC_517 inhibitor group mice were intraperitoneally injected with 5-aza-CdR inhibitor (0.2 mg/mL) and DC_517 inhibitor (1.7 μM) 200 µL respectively, administered once every 2 days, starting from week 5 and lasting for 4 weeks. The control group, model group, and JPZS group were intraperitoneally injected with an equal volume of physiological saline. After 8 weeks of treatment, fresh blood samples were collected from the orbit and fresh spleen for further analysis. Initially, there were 8 mice in each group. Due to insufficient tissue quantity, technical limitations or outlier elimination in some samples during detection, the actual n value of different experiments was different. All reported n values represent the number of independent biological repeats actually included in statistics. All animal procedures were approved by the Animal Ethics Committee of Anhui University of Chinese Medicine (AHUCM-mouse-2022130).

### Flow cytometry assay

To detect Th17 cells, 100 μL of fresh mouse whole blood or a suspension containing 1×10^6^ single-spleen-cells were taken and placed in a flow cytometry tube. Monoclonal antibodies such as CD4 (BioLegend, 100510) and IL-17A (BioLegend, 506903) were added, vortexed, and stained in dark at 4°C for 15 min. Following staining, 1 mL of hemolysin was added and incubated in the dark for 15 minutes. To detect Treg cells, 100 μL of fresh mouse whole blood or a suspension containing 1×10^6^ single-spleen-cells were taken and placed in a flow cytometry tube. Monoclonal antibodies such as CD4 (BioLegend, 100510), CD25 (BioLegend, 102027), and *Foxp3* (BioLegend, 320011) were added, vortexed, and stained in dark at 4°C for 15 min. After staining, 1 mL of hemolysin was added and incubated for 15 min in the dark (27, 28). Cell detection was performed using CytoFLEX flow cytometry (Beckman Coulter, Brea, CytoFLEX, USA), and the data were analyzed with FlowJo software (BD Biosciences, Franklin Lakes, NJ, USA).

### Extraction of CD4^+^ T cells from mouse spleen

Suspensions of mouse splenocytes were prepared under aseptic conditions. Subsequently, CD4^+^ T cells were isolated from the freshly prepared splenocyte suspension using the magnetic-activated cell sorting method (Miltenyi, Germany, 130-104-454), following a negative selection strategy as per the manufacturer’s instructions. Isolated CD4^+^ T cells were collected and preserved for subsequent analyses, including quantitative real-time PCR (qPCR), western blotting (WB), flow cytometry, and measurement of DNA methylation levels.

### DNA extraction and DNA methylation analysis

Subsequent methylation analysis of the Foxp3 gene was performed on CD4+T cells from five experimental groups: Control (n=4 mice), Model and Model+JPZS (n=8 each), and Model+5-Aza-CdR and Model+DC-517 (n=5 each). Genomic DNA was extracted from CD4+T cells using the TIANamp Genomic DNA Kit (Tiangen Biotech, China; Cat. DP304), followed by bisulfite conversion with the EZ DNA Methylation-Gold™ Kit (Zymo Research, USA) per manufacturer protocols. Methylation levels of cytosine-phosphate-guanine (CpG) dinucleotides within the *Foxp3* transcription start site region were quantified via MethylTarget™ targeted bisulfite sequencing (Genesky Biotech, China) using primers designed with FastTarget V4.1 software (Genesky, China): *Foxp3-TSDR* forward 5′-TTGGGTTTTTTTGGTATTTAAGA-3′ and reverse 5′-AAATCTACATCTAAACCCTATTATCACAA-3′. Bisulfite-converted DNA underwent PCR amplification followed by high-throughput sequencing on the Illumina HiSeq 2500 platform (Illumina, USA) with 150-bp paired-end reads.

### Total RNA isolation and qPCR

Total RNA was isolated from CD4^+^ T cells using a TRIzol kit (Life Technologies, cat. 15596018). cDNA was generated using the PrimeScript™RT reagent Kit with gDNA Eraser (TaKaRa, cat. RR047A). qPCR was performed using the Novostart SYBR qPCR SuperMix Plus Kit (Novoprotein, E096-01B) and detected using a PikoReal PCR cycler (Thermo Fisher Scientific, PIKOREAL 96) (29). The primer sequences were as follows: *Foxp3*, primer-F 5′-TGCCCATCTCTGTCTCAATC-3′ and primer-R 5′-GAAGTTGCTGCTTTAGGTGG-3′; β-actin, primer-F 5′-AGTGTGACGTTGACATCCGT-3′ and primer-R 5′-TGCTAGGAGCCAGAGCAGTA-3′; Dnmt1, primer-F 5′-ACAGTGACACCCTTTCAGTT-3′ and primer-R 5′-TCTGTGTCTACAACTCTGCG-3′; *RORγt*, primer-F 5′-TGGCACACAATCTCTTCCTT-3′ and primer-R 5′-CGGTCCTCTGCTTCTCTTAG-3′.

### Total cell protein extraction and western blotting assay

Using RIPA lysis buffer (Beyotime, P0013B), CD4^+^ T cells were lysed and centrifuged at 14,000 rpm for 15 minutes to extract total protein. Protein levels were measured with the BCA Protein Assay Kit (Beyotime, P00150). Gels were created following the guidelines provided by the SDS-PAGE Gel Preparation Kit (Beyotime, PG111). Using SDS-polyacrylamide gels (10% SDS), proteins were separated and transferred onto polyvinylidene fluoride membranes. After transferring, the membranes were treated with a 10% skim milk solution in Tris-buffered saline with Tween and then exposed to primary antibodies targeting Dnmt1 (1:1000, Bioss Inc, bs-0678R) at 4°C for 12 hours. A horseradish peroxidase-conjugated secondary antibody (1:20000; Zsbio) was then applied to the membranes for 1 hour. GAPDH antibody (1:5000; Zsbio) was used as the reference protein for normalization. After washing with TTBS, signals were detected using an ECL detection kit (Thermo, 340958). The obtained data were analyzed using ImageJ software.

### Detection of dsDNA, complement 3 (C3), cytokine and enzyme concentrations

Following the manufacturer’s guidelines, serum levels of IL-17, IL-23, TGF-β1, and IFN-γ, anti-dsDNA antibodies, Immunoglobulin G (IgG) and C3 were measured using an enzyme-linked immunosorbent assay (ELISA) kit. The IL-17 (JYM0554Mo), IL-23 (JYM0394Mo), TGF-β1 (JYM0215Mo), IFN-γ (JYM0540Mo), anti-dsDNA antibodies (JYM1061Mo), IgG (JYM0031Mo) and C3 antibodies (JYM0293Mo) ELISA kits were purchased from Wuhan Gene Beauty Biotechnology Co., Ltd. The absorbance values were measured at a wavelength of 450 nm using a microplate reader (Rayto RT-6000, China). To determine Dnmt1, Dnmt3A, and Dnmt3B enzyme activity, nuclear proteins from CD4^+^ T cells were purified using a nuclear extraction kit (Epigentek, Brooklyn, NY, USA). EpiQuik DNMT assay kit (Epigentek, Brooklyn, NY, USA) was used to assess the enzyme activity of Dnmt1, Dnmt3A, and Dnmt3B.

### Immunofluorescence

Mouse spleen tissues (4 μM) were sectioned and subjected to immunofluorescence staining. Sections were treated with mouse anti-FOXP3 (Santa, sc-53876) and anti-IL-17A antibodies (Bioss, bs-2140R) and incubated for 60 minutes at 37°C. The sections were then incubated for 30 minutes in a dark 37°C incubator using immunofluorescence secondary antibodies (goat anti-rabbit IgG [FITC] and goat anti-mouse IgG [CY3]/1:400). Nuclei were counterstained with DAPI. The images were captured using a panoramic MIDI scanner (3DHISTECH, Budapest, Hungary).

### Statistical analysis

Statistical analyses were performed using IBM SPSS software (version 26.0; IBM, Armonk, NY, USA), and graphs were created using GraphPad Prism 8.0 software (GraphPad Software Inc., San Diego, CA, USA). Student’s t-tests were used to compare data between two groups. A one-way analysis of variance followed by Tukey’s *post-hoc* test was used to assess statistical significance among multiple groups. Statistical significance was set at *P*<0.05.

## Results

### Component analysis of JPZS

The chromatogram of the mixed standard and JPZS is shown in **Figure 1**. The chemical components of the JPZS samples were analyzed using HPLC-MS. In a negative ion mode, we could detect the existence of gallic acid (found at 169.1 m/z), acteoside (623.15 m/z), catechin (289.1 m/z), hyperoside (463.05 m/z), rutin (609.1 m/z), quercetin (301 m/z), tiliroside (593.1 m/z), kaempferol (284.85 m/z), formononetin (267.05 m/z), astragaloside A (829.25 m/z), dioscin (913.4 m/z), and pachymic acid (527.35 m/z). In a positive ion mode, we could detect the existence of 5-hydroxymethylfurfural (found at 127.1 m/z), atractylenolide III (249.1 m/z), atractylone (217.25 m/z), and atractylodin (183.15 m/z).While this analysis confirmed the presence of key bioactive constituents previously associated with immunomodulation, future studies employing untargeted metabolomic approaches (e.g., UPLC-QTOF-MS/MS) will be undertaken to comprehensively characterize the entire chemical profile of JPZS, including potential minor yet potent components.

### JPZS improves disease development in MRL/lpr mice

**Figure 2:**
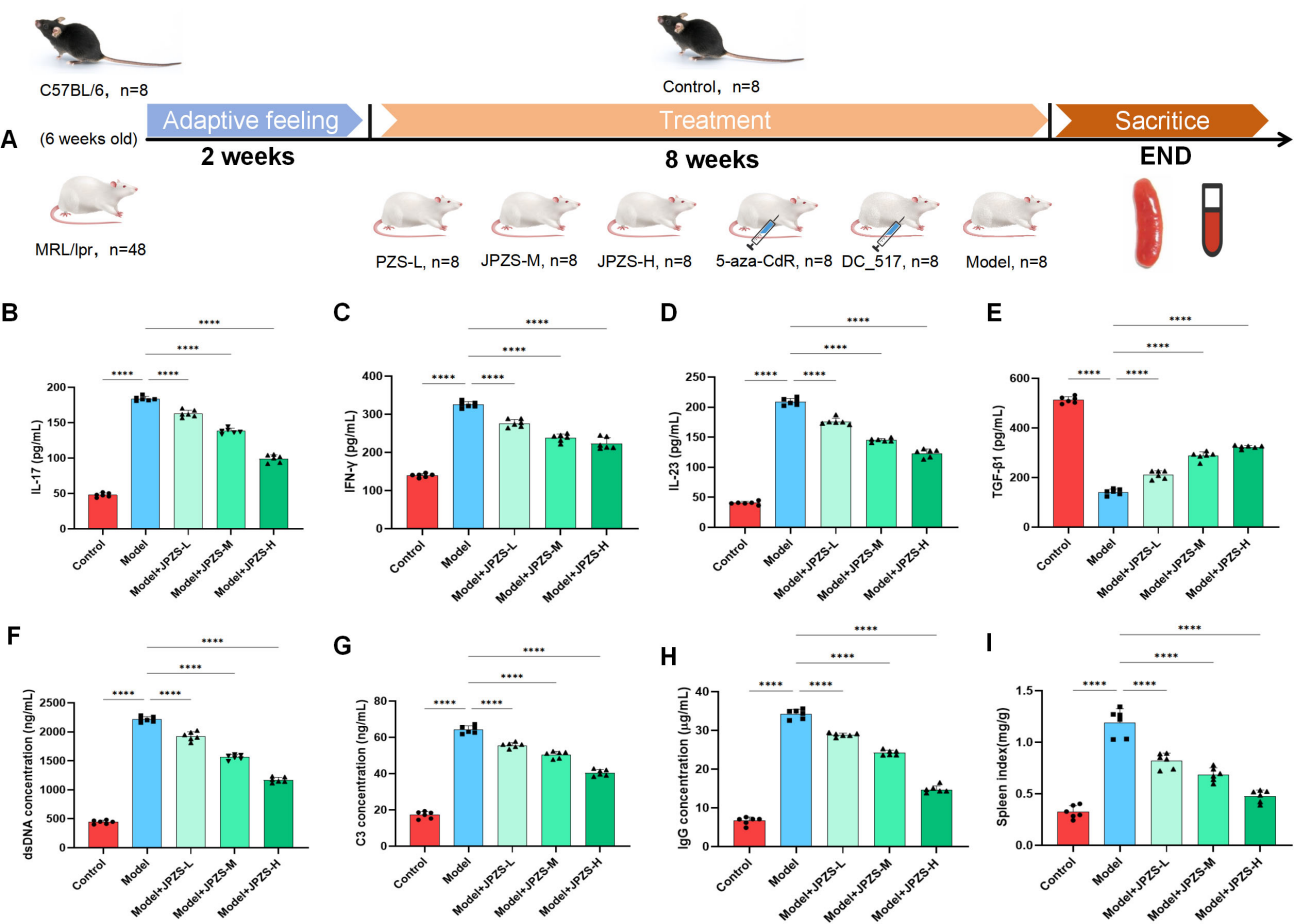
The JPZS treatment improves MRL/lpr mice disease development. **(A–D)** The effect of JPZS on the levels of serum cytokines TGF-β, IFN-γ, IL-17, and IL-23 in each group of mice. **(E)** The effect of JPZS on serum dsDNA levels in various groups of mice. **(F)** The effect of JPZS on serum C3 levels in each group of mice. **(G)** The effect of JPZS on serum anti-IgG levels in various groups of mice. **(H)** The effect of JPZS on the spleen index of mice in each group. **(I)** The effect of JPZS on the spleen index of mice in each group (Groups are the same as **B-E**). ****P<0.0001.

Female MRL/lpr mice (6-weeks-old) were acclimatized for 2 weeks and randomly allocated to six groups (n=8/group).MRL/lpr mice were administered saline, JPZS at low/medium/high doses (6.305/12.61/25.22 g/kg/day), 5-aza-CdR (0.2 mg/mL intraperitoneal injection), or DC_517 (1.7 μM intraperitoneal injection) from Week 8 to Week 16 (**Figure 2A**), with C57BL/6 mice serving as the healthy control group. All mice were sacrificed at Week 8 for serum and spleen analyses. To investigate the effect of JPZS on the disease progression of MRL/lpr mice, we measured the levels of cytokines (TGF-β, IFN-γ, IL-17, and IL-23), dsDNA, IgG, and C3 in the mouse serum. TGF-β levels declined significantly in the model group (*P <*0.01), while IFN-γ, IL-17, and IL-23 levels increased significantly (*P <*0.01) (**Figures 2B–E**). TGF-β levels increased significantly following JPZS intervention (*P <*0.01), while IFN-γ, IL-17, and IL-23 levels decreased significantly (*P <*0.01). Compared to the normal control group, the spleen index of the model group was significantly higher, but decreased significantly after the intervention (*P <*0.01) (**Figure 2I**). Immune system disorders in SLE are characterized by increased dsDNA and IgG levels and decreased C3 levels (30, 31). **Figures 2F, H** show that the levels of dsDNA and IgG in the serum of the model group were significantly elevated (*P*<0.01). After JPZS intervention, both dsDNA and IgG levels decreased significantly in a dose-dependent manner (*P*<0.01). As shown in **Figure 2G**, C3 levels in the serum of the model group were significantly reduced. Following JPZS intervention, C3 levels significantly increased in a dose-dependent manner (*P*<0.01). Compared to the normal control group, the spleen index of the model group was significantly elevated; however, after JPZS intervention, it decreased significantly in a dose-dependent manner (*P*<0.01) (**Figure 2I**). These data confirm that JPZS effectively alleviated disease progression in MRL/lpr mice, with JPZS-H demonstrating the most significant effects.

### Effects of JPZS treatment on Th17 and Treg cell balance

**Figure 3:**
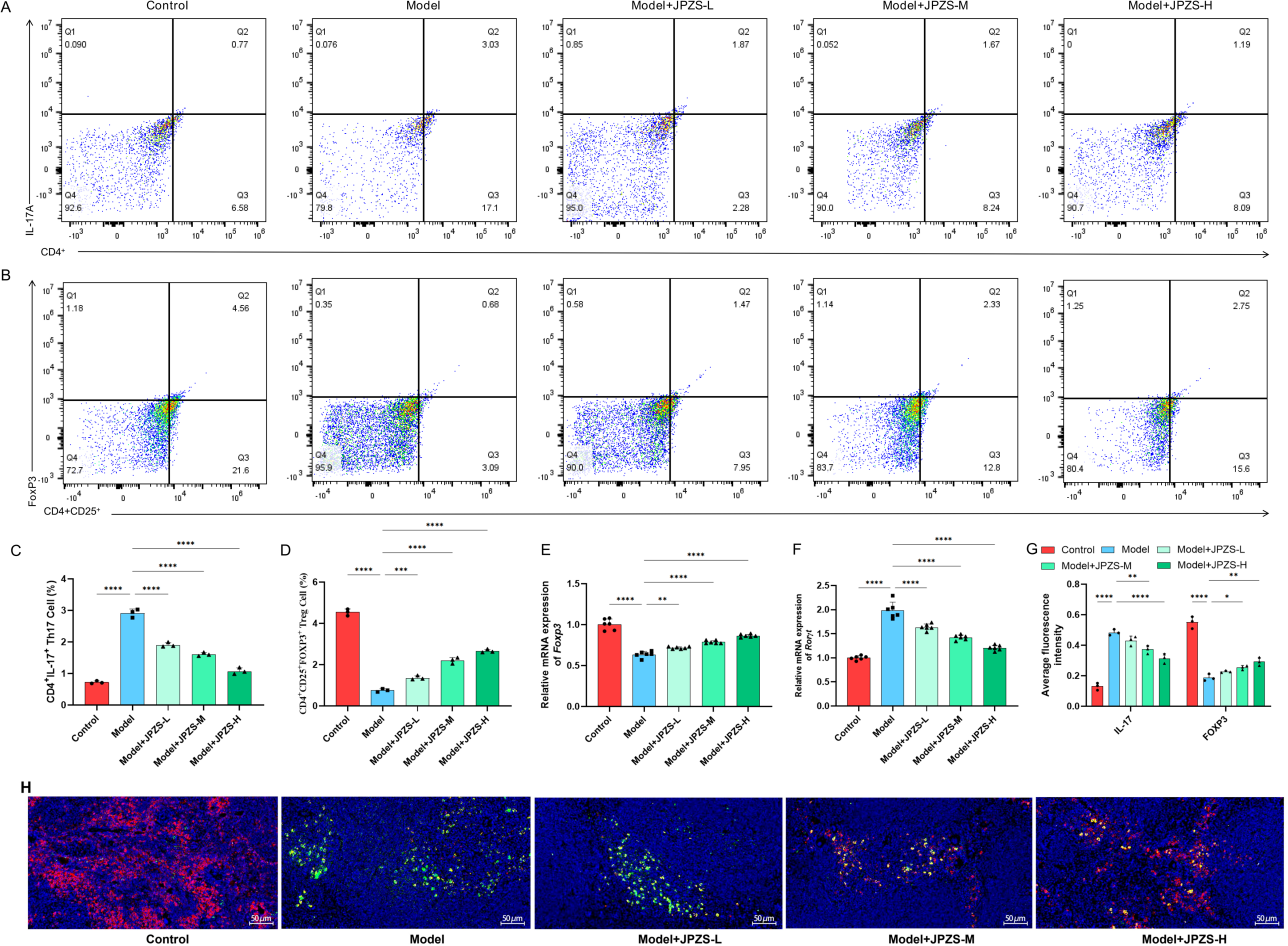
Effects of JPZS treatment on Th17 and Treg cell balance. **(A, B)** The effect of JPZS on the distribution of Tregs and Th17 cell subsets in peripheral blood CD4^+^ T cells of MRL/lpr mice indicated by flow cytometry analysis. **(C)** Quantitative histogram of Th17 cell frequency. **(D)** Tregs cell frequency quantification bar chart. **(E, F)** The effect of JPZS on the transcription levels of Foxp3 and RORγt mRNA in the peripheral blood CD4^+^ T cells of MRL/lpr mice. **(G)** Immunofluorescence quantitative bar charts of FOXP3 and IL-17A in the spleen of mice in each group. **(H)** Immunofluorescence staining of spleen FOXP3 and IL-17A in each group of mice (scale: 50 μm; IL-17A: green; FOXP3: red). ****P<0.0001, ***P<0.001, **P<0.01, *P<0.05.

The Th17/Treg ratio can directly reflect the autoimmune status of SLE (32, 33). As demonstrated in **Figures 3A–D**, the proportion of Th17 cells in the spleen of the model group mice significantly increased, while the proportion of Treg cells significantly decreased (*P <*0.01); JPZS intervention resulted in a dose-dependent reduction in the proportion of Th17 cells and an increase in the proportion of Treg cells (*P <*0.01). We further observed FOXP3/IL-17A in the spleen using an immunofluorescence assay (**Figures 3G, H**) and found that it was consistent with the flow cytometry results. The expression of the transcription factors *RORγt* and *Foxp3* mRNA in CD4^+^ T cells was further analyzed based on these findings. We observed that mRNA levels of *RORγt* in CD4^+^ T cells from the model group were elevated compared to the control group, while the transcription level of *Foxp3* mRNA was significantly reduced (*P <*0.01). After JPZS intervention, these changes were reversed in a dose-dependent manner (**Figures 3E, F**). As shown in **Figures 1**, **2**, the imbalance in the Treg/Th17 ratio is associated with disease progression in SLE, and JPZS effectively alleviates disease progression in MRL/lpr mice. Additionally, the effects of JPZS were dose-dependent, with JPZS-H demonstrating the most significant improvement in disease condition; therefore, subsequent studies will utilize high doses of JPZS for further investigation.

### DNMT1-mediated Foxp3-TSDR methylation and JPZS reduction

**Figure 4:**
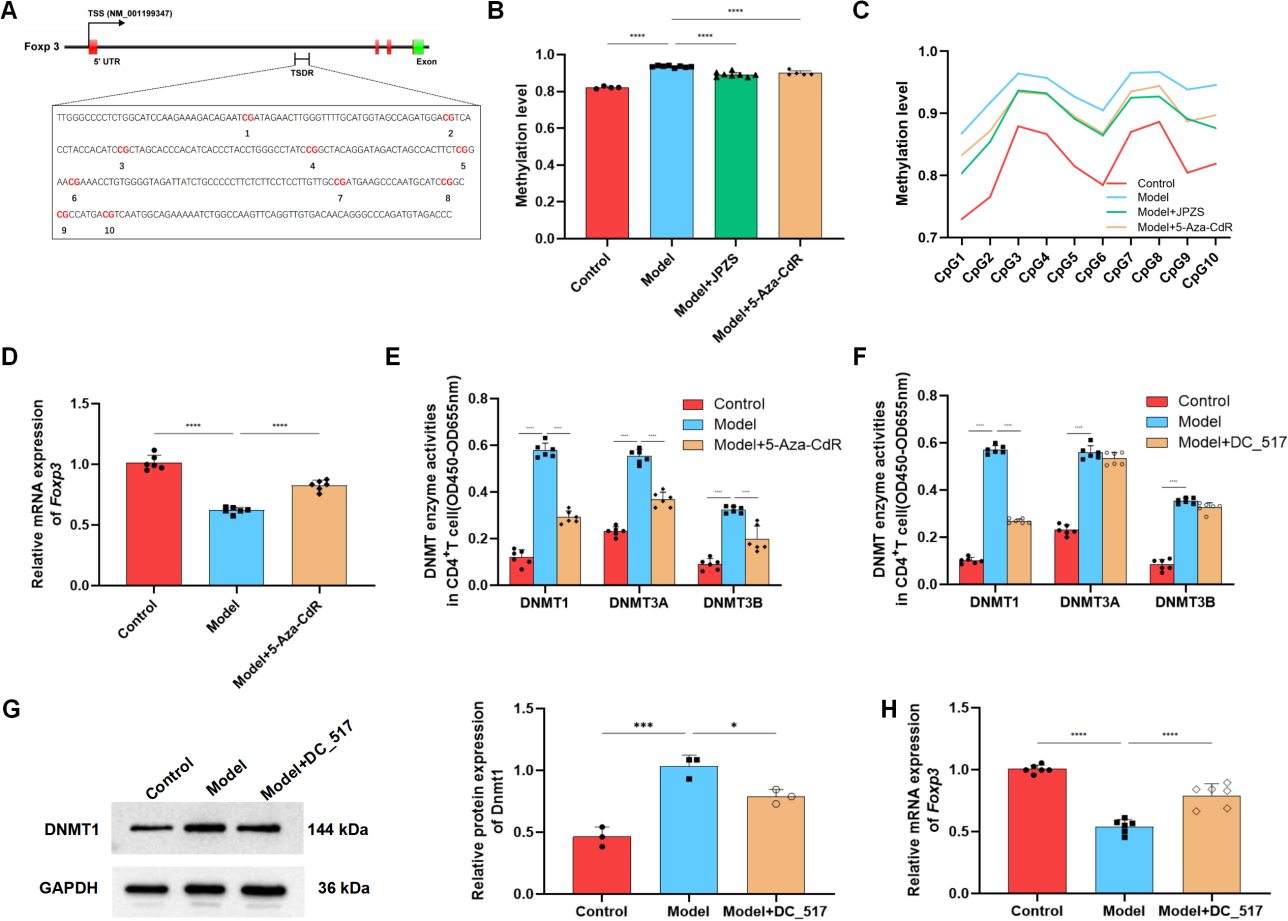
DNA methylation levels of *Foxp3* in the CD4^+^ T cells of MRL/lpr mice and the reducing effect of JPZS. **(A)** CPG islands of *Foxp3*-*TSDR* in CD4^+^ T cells of MRL/lpr mice. **(B)** The effects of JPZS and 5-aza-CdR on the *Foxp3* methylation levels in CD4^+^ T cells. **(C)** The effect of JPZS and 5-aza-CdR on the methylation levels of 10 CPG islands in the CD4^+^ T cells *Foxp3*. **(D)** The transcription level of *Foxp3* mRNA after 5-aza-CdR treatment. **(E)** Methyltransferase Dnmt1, Dnmt3a, and Dnmt3b activities were noted in the CD4^+^ T cells treated with 5-aza-CdR. **(F)** Methyltransferase Dnmt1, Dnmt3a, and Dnmt3b activities were observed in the CD4^+^ T cells treated with DC_517. **(G)** The expression level of the Dnmt1 protein after DC_517 treatment. **(H)** The transcription level of *Foxp3* mRNA after DC_517 treatment. ****P<0.0001, ***P<0.001, *P<0.05.

To investigate the DNA methylation reduction effect of JPZS on the *TSDR* region of *Foxp3*, we used Methylation-specific PCR (MSP) sequencing to analyze the average *Foxp3* methylation levels in the CD4^+^ T cells in each group. There were 10 CpG loci in the TSDR region of *Foxp3* in the CD4^+^ T cells of MRL/lpr mice in the model group (**Figure 4A**). As demonstrated in **Figure 4B**, the level of *Foxp3* methylation in the peripheral blood CD4^+^ T cells of the model group mice significantly increased, while after intervention with JPZS and 5-aza-CdR (DNMT inhibitor), the *Foxp3* methylation level significantly decreased (*P <*0.01). **Figure 4C** also indicates that the methylation levels of the 10 CpG sites in the TSDR region of the *Foxp3* gene in the model group were significantly increased, while the methylation levels of the 10 CpG sites were significantly downregulated after JPZS and 5-aza-CdR intervention (*P <*0.01). We found that the transcription level of *Foxp3* mRNA was significantly upregulated after 5-aza-CdR intervention (*P <*0.01) (**Figure 4D**). The above experiments indicate that JPZS has a reducing effect on *Foxp3*-*TSDR* methylation, whereas the regulation of DNMT can mediate *Foxp3*-*TSDR* demethylation.

Mammals have three types of DNMTs with catalytic activity: DNMT1, DNMT3A, and DNMT3B. We further investigated whether DNMT is dependent on the demethylation of *Foxp3* in CD4^+^ T cells. First, we determined the enzymatic activity of DNMT1, DNMT3A, and DNMT3B. As demonstrated in **Figure 4E**, the activity of all three enzymes in the model group was upregulated, whereas the activity was significantly reduced after 5-aza-CdR intervention (*P <*0.01). DC_517 is a Dnmt1-specific inhibitor, and after DC_517 intervention, only DNMT1 enzyme activity significantly decreased (*P <*0.01) (**Figure 4F**). We further investigated and found that after DC_517 intervention, the expression of DNMT1 protein was significantly reduced (*P <*0.01) (**Figure 4G**), and the transcription level of *Foxp3* mRNA was significantly upregulated (*P <*0.01) (**Figure 4H**).

**Figure 5:**
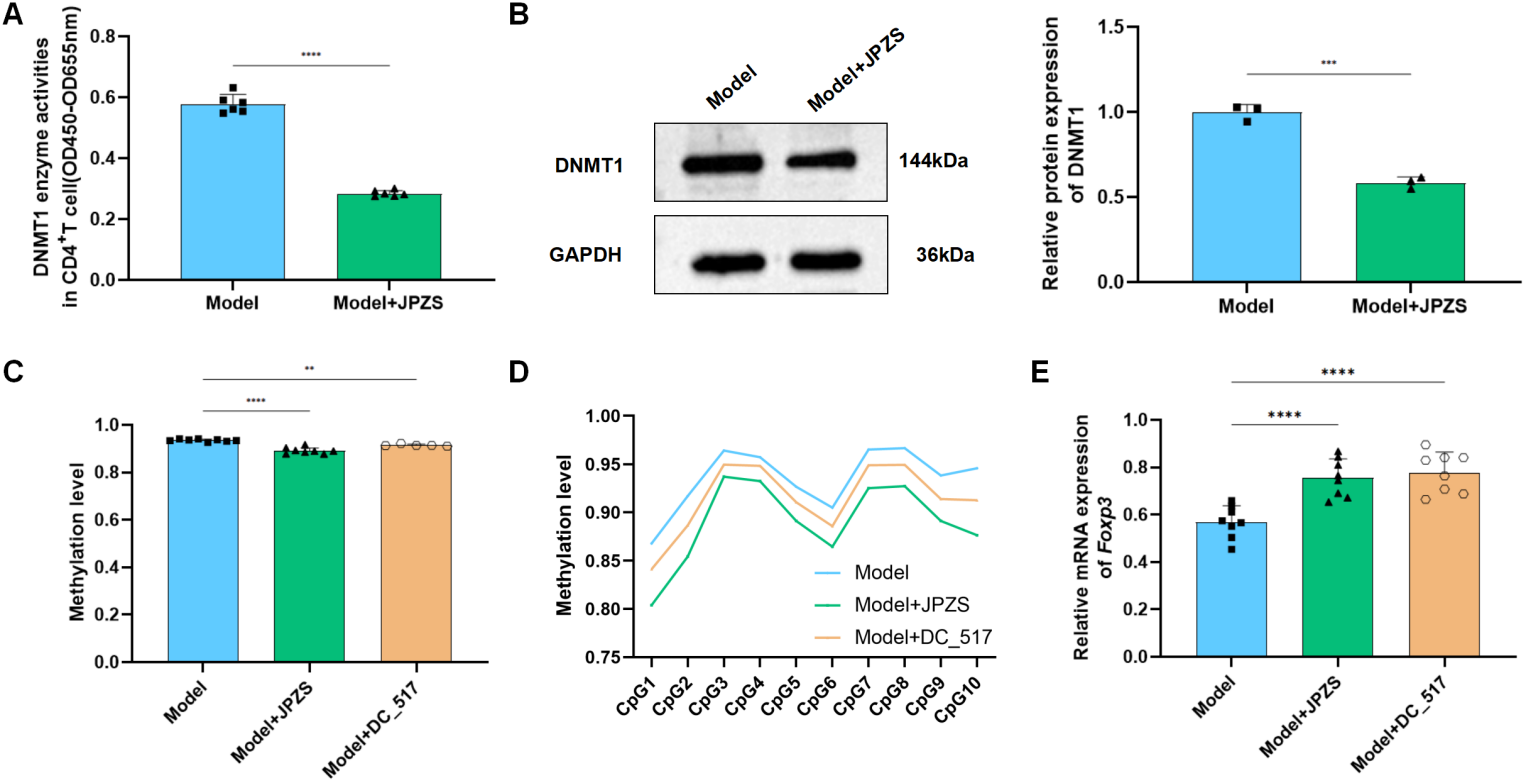
DNMT1-mediated *Foxp3*-*TSDR* methylation and reduction of JPZS. **(A, B)** The effect of JPZS on Dnmt1 activity and protein expression levels. **(C)** The effects of JPZS and DC_517 on *Foxp3* methylation levels in CD4^+^ T cells. **(D)** The effects of JPZS and DC_517 on the methylation levels of 10 CpG islands in CD4^+^ T cells *Foxp3*. **(E)** The effect of JPZS and DC_517 on the transcription level of *Foxp3* mRNA in the peripheral blood CD4^+^ T cells of MRL/lpr mice. ****P<0.0001, ***P<0.001, **P<0.01.

After confirming that *Foxp3*-*TSDR* methylation primarily relies on Dnmt1 mediation, we further examined whether the effect of JPZS on *Foxp3*-*TSDR* methylation also depends on Dnmt1 mediation. **Figures 5A, B** show that after JPZS intervention, Dnmt1 activity and DNMT1 protein expression levels were significantly lower (*P* <0.01). *Foxp3*-*TSDR* methylation levels decreased significantly (**Figure 5C**) following DC_517 intervention (**Figure 5C**). Simultaneously, the methylation levels of CpG1, CpG3, CpG7, CpG9, and CpG10 at the 10 CpG sites of *Foxp3*-*TSDR* were significantly reduced (*P <*0.05; *P <*0.01) (**Figure 5D**), *Foxp3* mRNA level significantly upregulated (*P <*0.01) (**Figure 5E**), which was consistent with the trend observed after JPZS intervention. These results indicate that JPZS reduces *Foxp3*-*TSDR* methylation, mainly through Dnmt1 mediation.

### JPZS promotes Treg/Th17 balance by inhibiting DNMT1-mediated Foxp3 methylation

**Figure 6:**
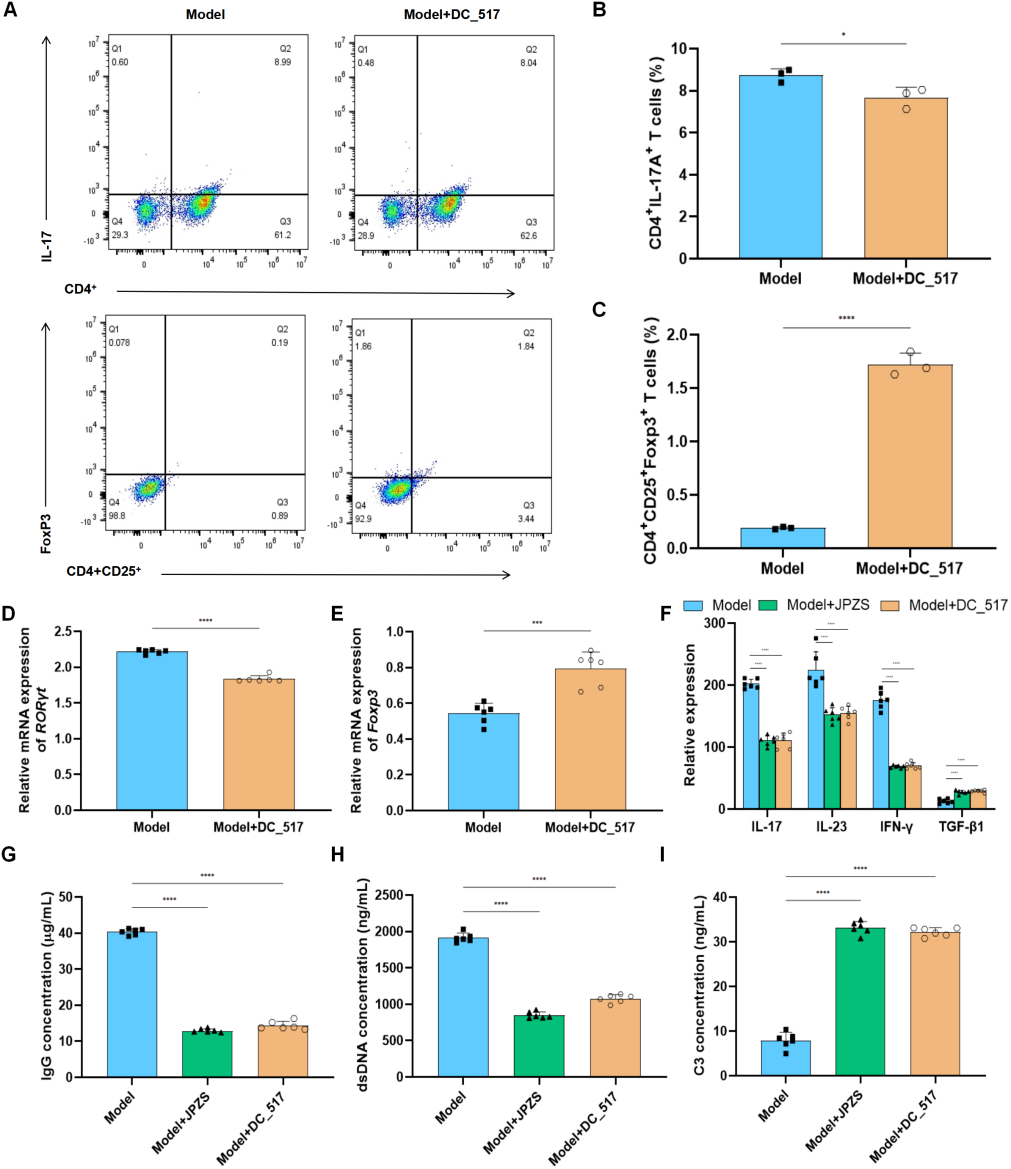
JPZS promotes Treg/Th17 balance by inhibiting DNMT1-mediated *Foxp3* methylation. **(A)** The frequency of Th17 cells and Treg cells in splenocytes was denoted by flow cytometry analyses. **(B, C)** The effects of JPZS and DC_517 on the transcription levels of *Foxp3* and *RORγt* mRNA in CD4^+^ T cells. **(D)** Effects of JPZS and DC_517 on serum cytokine levels in MRL/lpr mice. **(G–I)** The effects of JPZS and DC_517 on the levels of IgG, dsDNA, and C3 in the serum of MRL/lpr mice. ****P<0.0001, ***P<0.001, *P<0.05.

We investigated whether JPZS promoted the Treg/Th17 balance by inhibiting DNMT1-mediated *Foxp3* methylation. In **Figures 6A–C**, compared with the model group, the proportion of Th17 cells to CD4^+^ T cells decreased in the model+DC_517 group, while the proportion of Treg cells to CD4^+^ CD25^+^ T cells increased (*P <*0.05). The above experimental results are consistent with the effect of the model+JPZS group on the distribution of Tregs and Th17 cell subsets in CD4^+^ T cells of MRL/lpr mice in **Figures 3A–C**.

**Figures 6D, E** shows that, in comparison with the model group, the transcription level of *RORγt* mRNA was significantly reduced in the model+DC_517 group peripheral blood CD4^+^ T cells, while *Foxp3* mRNA was significantly increased (*P <*0.05). These results are consistent effect of JPZS on the transcription levels of *Foxp3* and *RORγt* mRNA in the peripheral blood CD4^+^ T cells of MRL/lpr mice in **Figures 3F, G**.

As shown in **Figure 6F**, the serum concentrations of TGF-β1 were higher in the model+JPZS and model+DC_517 groups of mice compared to the model group (*P <*0.01), whereas the levels of IFN-γ, IL-17, and IL-23 were lower (*P <*0.01). This indicate that JPZS and DC_517 have similar effects on the levels of cytokines in the serum of MRL/lpr mice, both promoting the production of anti-inflammatory factors and inhibiting inflammatory responses.

As shown in **Figures 6G–I**, compared with the model group, the levels of IgG and dsDNA in the serum of MRL/lpr mice in the model+JPZS and model+DC_517 groups were significantly reduced (*P <*0.01), whereas the levels of C3 were significantly increased (*P <*0.01), indicating that both DC_517 and JPZS can improve disease activity in MRL/lpr mice by reducing IgG and dsDNA levels and increasing C3 levels.

## Discussion

The paradigm of SLE pathogenesis extends beyond a simple numerical imbalance in Th17/Treg populations to encompass profound epigenetically enforced immune dysregulation. Our findings elucidate a key pathological mechanism in the MRL/lpr model: heightened DNMT1 expression within CD4^+^ T cells is mechanistically linked to the facilitation of aberrant *Foxp3-TSDR* hypermethylation. This epigenetic silencing directly contributes to diminished *Foxp3* mRNA transcription levels and a consequent decline in the functional Treg population, thereby destabilizing immune tolerance. Critically, administration of JPZS effectively ameliorated disease progression in this murine model, correlating with a restored Treg/Th17 balance. Mechanistically, JPZS appears to exert this immunomodulatory effect, at least in part, by suppressing DNMT1 enzymatic activity and impeding Foxp3 hypermethylation, mirroring the effects observed with the specific DNMT1 inhibitor DC_517. This positions DNMT1-mediated epigenetic dysregulation of Foxp3 as a significant targetable pathway in SLE pathogenesis and highlights JPZS’s potential to intervene at this level.

SLE management often incorporates Traditional Chinese Medicine (TCM) (34, 35). A large-scale cohort study (n=10,462) has demonstrated that TCM significantly reduces the risk of end-stage renal disease (ESRD) by 76% (adjusted HR=0.24, 95% CI 0.07-0.80) and all-cause mortality by 30% (aHR=0.70, 95% CI 0.58-0.83) compared to non-TCM users (36). The JPZS formula, developed based on the “spleen-kidney tonification” principle, represents a targeted therapeutic strategy for SLE. Clinical evidence demonstrates that in an eight-week randomized controlled trial (n=60), JPZS can restore immune homeostasis in SLE patients. Specifically, this was manifested by the reestablishment of balance between regulatory Tregs and Th17 helper cells. The CD4+/CD8+ ratio increased from 0.93 ± 0.04 to 2.61 ± 0.04 (*P <*0.01), while Th17-associated cytokine levels decreased (25, 26). Research has shown that the herbal formula and its active constituents in JPZS are effective in addressing diseases associated with Treg/Th17 imbalance (24, 37, 38).

The therapeutic efficacy of JPZS observed in this study is likely attributable to the synergistic actions of its multiple bioactive constituents, as identified by our HPLC-MS analysis (**Figure 1**).

Notably, Astragaloside A, a major saponin from Astragalus membranaceus, has been consistently reported to promote Treg differentiation and *Foxp3* expression (29). Acteoside and other constituents from Rehmannia glutinosa possess documented DNMT inhibitory activity and anti-inflammatory properties, aligning with our findings of reduced *Foxp3-TSDR* methylation and suppressed inflammation (39, 40). Quercetin attenuated renal inflammation by inhibiting NLRP3 inflammasome activation and TGF-β/Smad3-mediated fibrosis (41, 42). Rutin significantly improved a variety of immune indicators, including red blood cell count, hemoglobin content, macrophage activity, lymphocyte proliferation and serum concentration of cytokines (43, 44). Kaempferol enhanced Treg suppressive function by stabilizing *Foxp3* through inhibition of miR-34a expression, as evidenced by upregulated *Foxp3*/IL-10/TGF-β and downregulated RORγt/IL-17 in T cells (45, 46). This confluence of actions from diverse components targeting DNMT1 activity, Foxp3 expression, Treg function, and Th17 polarization collectively underpins the ability of JPZS to rectify the Treg/Th17 imbalance and ameliorate SLE pathology in the MRL/lpr model. Future studies isolating individual compounds and assessing their specific contributions to the observed DNMT1 inhibition and epigenetic modulation are warranted.

The paradigm of SLE pathogenesis extends beyond Th17/Treg numerical imbalance to epigenetically enforced immune dysregulation (32, 47). Our work demonstrates that DNMT1-mediated *Foxp3* silencing imposes a developmental blockade on Treg precursors, thereby licensing pathological Th17 responses. JPZS counteracts this hierarchy through multidimensional immune resetting: (i) epigenetic rehabilitation of *Foxp3* loci, (ii) functional restoration of Treg suppressor capacity, and (iii) metabolic constraint of Th17 polarization. Unlike biologic agents that target singular pathways, JPZS represents a systems-level intervention capable of durable immune recalibration. This study focuses on the potential efficacy of JPZS drugs in restoring the balance between Tregs and Th17 cells by promoting the differentiation of Tregs.

*Foxp3*, the master transcription factor defining regulatory Treg lineage identity and function, plays a pivotal role in maintaining immune tolerance by suppressing aberrant effector responses, including those of Th17 cells (48–50). Crucially, the stable expression of *Foxp3* in Tregs is governed by epigenetic mechanisms, particularly DNA methylation within its locus. The Treg-specific demethylated region (*TSDR*) serves as an essential imprinting control element for sustaining *Foxp3* expression and Treg stability (51). Aberrant hypermethylation of the *Foxp3*-*TSDR*, often driven by dysregulated DNA methyltransferases like DNMT1, disrupts Treg differentiation and function, contributing to the pathogenic Th17/Treg imbalance observed in autoimmune diseases, including SLE (15, 52).

In this study, we identified a key pathological feature in MRL/lpr SLE mice: significantly elevated *Foxp3*-*TSDR* methylation in CD4+ T cells, concomitant with a profound downregulation of *Foxp3* mRNA and diminished Treg populations. Importantly, treatment with JPZS effectively reversed this epigenetic aberration, reducing *Foxp3*-*TSDR* methylation and restoring *Foxp3* expression. Mechanistically, our data demonstrate that JPZS achieves this by specifically suppressing DNMT1 enzymatic activity and protein expression, mirroring the effects of the DNMT1 inhibitor DC_517. This targeted inhibition of DNMT1-mediated *Foxp3* hypermethylation represents a core mechanism through which JPZS promotes Treg/Th17 rebalancing and ameliorates SLE pathology in this model. These findings significantly advance our understanding of TCM immunomodulation by revealing a precise epigenetic pathway (DNMT1-*Foxp3*-*TSDR*) underpinning JPZS’s therapeutic efficacy in SLE.

These findings establish JPZS as the first TCM formula proven to correct Treg/Th17 imbalance via DNMT1-Foxp3 axis modulation. Our data confirm the central role of DNMT1-mediated hypermethylation of the *Foxp3-TSDR* region in CD4+ T cells in disrupting Treg stability and contributing to SLE pathogenesis in MRL/lpr mice, consistent with prior research implicating DNMT1 in Treg-specific epigenetic dysregulation (53, 54). Crucially, JPZS intervention effectively reversed this pathological signature, mirroring the effects of the specific DNMT1 inhibitor DC_517. Both JPZS and DC_517 significantly reduced *Foxp3-TSDR* methylation, restored Foxp3 expression and Treg numbers, ameliorated the Th17/Treg imbalance, and improved key disease markers (reduced dsDNA/IgG/IFN-γ/IL-17/IL-23, increased C3/TGF-β). The mechanistic link was further solidified by JPZS’s specific downregulation of DNMT1 enzymatic activity and protein expression, demonstrating that its epigenetic action is predominantly mediated through DNMT1 inhibition.

These findings establish JPZS as the first TCM formula proven to correct Treg/Th17 imbalance via DNMT1-*Foxp3* axis modulation. The DNMT1-dependent demethylation of *Foxp3-TSDR* represents a precise epigenetic mechanism underlying its immunomodulatory efficacy, bridging traditional “spleen-kidney tonification” theory with contemporary epigenetics. Future studies should prioritize: (i) Validating this mechanism in human SLE CD4^+^ T cells, (ii) Identifying specific bioactive components within JPZS responsible for DNMT1 inhibition (e.g., Rehmannia-derived catalpol), and (iii) Exploring combinatorial therapies with conventional immunosuppressants to enhance clinical efficacy. Our work not only provides a scientific foundation for JPZS as a promising SLE therapeutic but also illuminates *DNMT1* as a druggable target for novel epigenetic-based interventions in autoimmunity.

## Conclusion

This study provides evidence that JPZS alleviates disease manifestations in MRL/lpr lupus-prone mice, potentially through mechanisms involving the modulation of Treg and Th17 cell dynamics. Our data suggest that JPZS may inhibit DNMT1-mediated hypermethylation of the *Foxp3-TSDR* region in CD4^+^ T cells, leading to increased Foxp3 expression and a trend toward Treg/Th17 rebalancing. These findings offer preliminary insights into the immunomodulatory actions of JPZS and shed light on a potential epigenetic regulatory mechanism, involving the DNMT1-*Foxp3* axis, that may contribute to its therapeutic effects in this experimental model of SLE. Further investigation, particularly in human systems, is warranted to confirm these mechanisms and assess their translational relevance.

## Data Availability

The datasets presented in this study can be found in online repositories. The names of the repository/repositories and accession number(s) can be found in the article/supplementary material.
